# Antibiotic resistance levels in soils from urban and rural land uses in Great Britain

**DOI:** 10.1099/acmi.0.000181

**Published:** 2020-11-23

**Authors:** Kieran Osbiston, Anne Oxbrough, Lorena Teresa Fernández-Martínez

**Affiliations:** ^1^​ Biology Department, Edge Hill University, Ormskirk, L39 4QP, UK

**Keywords:** antibiotic resistance, land use, agriculture, urban, semi-natural, soil

## Abstract

Although soil is one of the largest microbial diversity reservoirs, the processes that define its microbial community dynamics are not fully understood. Improving our understanding of the levels of antibiotic resistance in soils with different land uses in Great Britain is not only important for the protection of animal health (including humans), but also for gaining an insight into gene transfer levels in microbial communities. This study looked at the levels of antibiotic-resistant bacteria (ARB) able to survive inhibitory concentrations of chloramphenicol, erythromycin and vancomycin, as well as subinhibitory (10 µg ml^−1^) erythromycin concentrations. Soils from nine different sites across Great Britain with three distinct land uses (agricultural, urban and semi-natural) were sampled and the percentage of ARB was calculated for each site. Statistical analyses confirmed a significant difference in the level of ARB found in agricultural land compared to urban or semi-natural sites. The results also showed that resistance levels to vancomycin and chloramphenicol in the agricultural and urban sites sampled were significantly higher than those for erythromycin, whilst in semi-natural sites all three antibiotics show similar resistance levels. Finally, although the levels of resistance to a subinhibitory (10 µg ml^−1^) erythromycin concentration were significantly higher across land use types when compared to the levels of resistance to an inhibitory (20 µg ml^−1^) concentration, these were much less marked in soil from agricultural land compared to that from urban or semi-natural land use soil.

## Introduction

Antimicrobial resistance (AMR) is one of the most serious problems affecting global animal health (including humans). Although the increase in AMR is mostly driven by antibiotic and antimicrobial use, research suggests that the natural environment plays a key role in the wider spread of AMR [1–3]. While the use of antibiotics as growth promoters was phased out in the European Union between 1999 and 2006, their use in farming is still a major contributing factor to their release in large quantities to local ecosystems [4]. In addition, other anthropogenic activities in agriculture, including municipal wastewater irrigation [5] and the use of biosolids [6] and manure [7] in agricultural soils, can contribute to the spread of antibiotic-resistant bacteria (ARB) and antibiotic resistance genes (ARGs) in these soils.

Agricultural intensification, like many anthropogenic activities, impacts negatively on the environment [8]. Examples of these impacts include deforestation, soil degradation and pollution of water and soil. The preventative and therapeutic use of antibiotics in animal farming has been shown to contribute to an increase in AMR, via manure storage and the use of manure solids or wastewater as soil fertilizer [9–11]. The constant use of vast amounts of antibiotics in animal farming, leading to their release into the local environment, together with the increasing prevalence of ARB and ARGs, is a concern. While the use of antibiotics can increase the number of ARB, the relationship between ARB/ARGs, land use practices and antibiotic use in agriculture is poorly understood [12].

AMR is a natural phenomenon that predates the clinical use of antibiotics [13–15]. Although antibiotic resistance genes occur naturally in soils independently of anthropogenic activities [16], research suggests that their abundance in agricultural soils has been increasing since antibiotics were introduced for growth promotion purposes in animal farming [17], making their way into agricultural fields via manure application [16]. Manure application can transfer ARB and ARGs to soils, causing the expansion of antibiotic resistance reservoirs in comparison with non-manured soils [18–21].

The ARGs that confer resistance to antibiotics are currently considered to be environmental contaminants [22, 23]. Major sources of ARG pollution include animal-derived faeces and manure entering the environment via direct soil application [18, 24–30]. Antibiotics used in farm animals can accumulate in the soil and consequently spread as fertilizer on the farmland at low concentrations [31], but the effects of these low antibiotic concentrations on selection for resistant bacteria in the environment or specific bacterial community responses remain uncertain [29, 32, 33].

Although AMR levels in land used for agricultural purposes have been relatively well studied, not much is known about urban or more natural soil environments. It is important to consider that the increase in AMR in clinics could also be linked to horizontal gene transfer from natural ARG reservoirs from these understudied environments [34]. In order to define the level of anthropogenic impact in different land uses, AMR levels in semi-natural soil environments must be established [35].

Antibiotics commonly used in medicine include chloramphenicol and erythromycin. Although both antibiotics impede bacterial growth by inhibiting protein synthesis, the resistance mechanisms for them differ [36, 37]. Erythromycin is a macrolide, one the most common antibiotic classes routinely used in both human and veterinary medicine. Erythromycin was the first macrolide antibiotic used clinically to treat human infections [38] and it is still commonly prescribed due to its wide-spectrum activity against Gram-positive and some Gram-negative bacteria [39]. Vancomycin is a last-resort clinical antibiotic that blocks cell wall biosynthesis [40]. Resistance to vancomycin can arise through the modification of cell wall precursors [41, 42]. These three antibiotics are naturally produced by soil bacteria [43] and therefore natural levels of resistance to all three drugs would be expected to be present in soil environments independently of anthropogenic activities.

The objective of this study was to evaluate the levels of bacteria resistant to chloramphenicol, erythromycin and vancomycin present in soils across Great Britain with distinct land uses and to establish whether there is a relationship between resistance level and land use type, particularly regarding human activity. This study also aimed to determine the effect of differing concentrations on one particular antibiotic, erythromycin, on the number of ARB. Erythromycin at subinhibitory and inhibitory concentrations was chosen due to its historical use in veterinary practice and its stability in soil environments.

## Methods

### Sampling sites

**Fig. 1.**
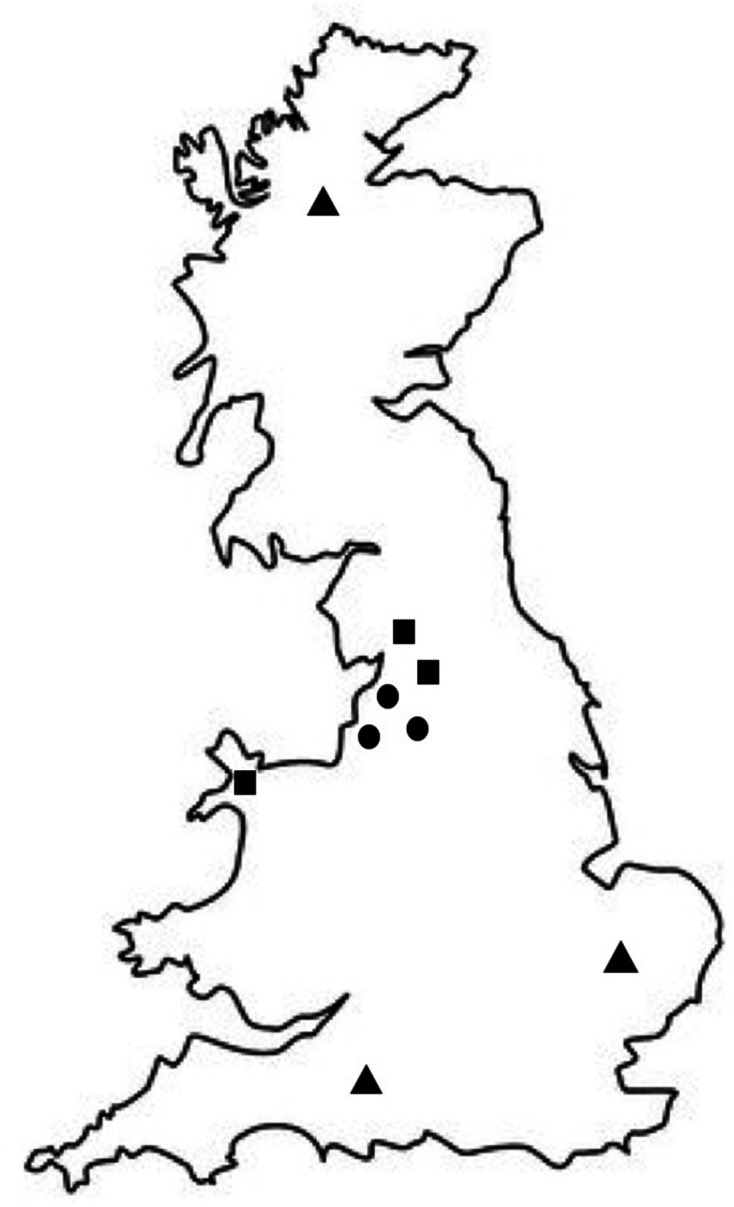
Locations of the nine sampling sites across Great Britain. Circles represent public parks (urban), squares represent animal farming land (agricultural) and triangles represent semi-natural sites.

Samples were collected from nine sites across Great Britain (Fig. 1). The selected sites represent three distinct land uses: ‘urban’, comprising improved grasslands in public parks embedded within urban areas that have a high daily volume of visitors; ‘agricultural’, comprising improved grasslands with intensive cattle grazing, recently limed and spread with cow manure, but less human access; and, ‘semi-natural’, comprising mature Scots pine plantation forests in remote areas with little human or captive animal interaction.

### Soil sampling

Six 10 g soil samples were taken from each of the 9 sites in this study, giving a total of 54 samples. The six sampling points within each site were selected in homogenous habitat areas and away from the edges. Soil was collected from the top 10 cm soil layer by using a small trowel, which was washed with sterile water and 70 % ethanol in-between sampling points. Soil samples were immediately placed in 15 ml sterile Falcon tubes labelled with the site name and the GPS location. Samples were kept refrigerated in a cooler bag during sampling and stored at 4 °C as soon as possible.

### Testing of soil samples for antibiotic resistance strains

The antibiotics we selected for study – chloramphenicol, erythromycin and vancomycin – are critically important in human medicine [44], belong to different antimicrobial classes and therefore possess different resistance mechanisms, and cover a wide range of bacterial hosts [45, 46].

One gram from each soil sample collected was used to generate 10^0^ to 10^−3^ dilutions. Twenty microlitres of each dilution was plated in triplicate onto R2A plates containing nystatin (50 µg ml^−1^) as a control or nystatin (50 µg ml^−1^) plus one of the following antibiotics: chloramphenicol (20 µg ml^−1^), erythromycin (10 µg ml^−1^), erythromycin (20 µg ml^−1^) and vancomycin (20 µg ml^−1^). This process was repeated five times with 1 g of soil from each sampling point.

R2A plates were incubated for 7 days at 30 °C. Nystatin (50 µg ml^−1^) was added to all plates to prevent fungal growth. The number of ARB c.f.u. ml^−1^ present was calculated for both control and antibiotic-containing plates (Table S1, available in the online version of this article).

### Statistical analysis

The percentage of ARB c.f.u. ml^−1^ on plates containing antibiotics with respect to the total number of c.f.u. ml^−1^ present in their control plates was measured for each antibiotic and/or concentration of antibiotic. Then, the mean percentage of ARB was calculated using the percentage of ARB c.f.u. ml^−1^ values obtained for each dilution on each of the five replicates taken per sample. To determine whether land use and antibiotic type influenced the number of culturable antibiotic strains, percentage ARB was tested using generalized linear mixed models (GLMMs) with land use and antibiotic (at 20 µg ml^−1^ concentration) as fixed factors and site as a random factor. ARB was centred around zero to improve model fit and a Gamma distribution was used for zero bounded data. Where fixed factors in the final model were significant these were explored graphically. Final models were checked to ensure that they met the assumptions of the GLMMs following procedures outlined elsewhere [47].

To determine whether antibiotic concentration influences the number of culturable antibiotic strains, percentage ARB was tested using GLMMs with concentration of erythromycin and land use type as fixed factors, and site as a random factor. Although land use type was not of primary interest here, its inclusion allows us to test the interaction between concentration and land use. ARB was centred around zero, a Gamma distribution was used and models were checked and tested for significance as described above for the GLMMs. All analyses were carried out in the R statistical programme (version 4.0.0) [48]. GLMM used the ‘lmer’ function in the *lme4* package [49]. Significance was tested using the ‘Anova’ function in the *car* package [50].

## Results

### Levels of ARB in soil depend on land use and resistance differs between tested antibiotics

**Fig. 2.**
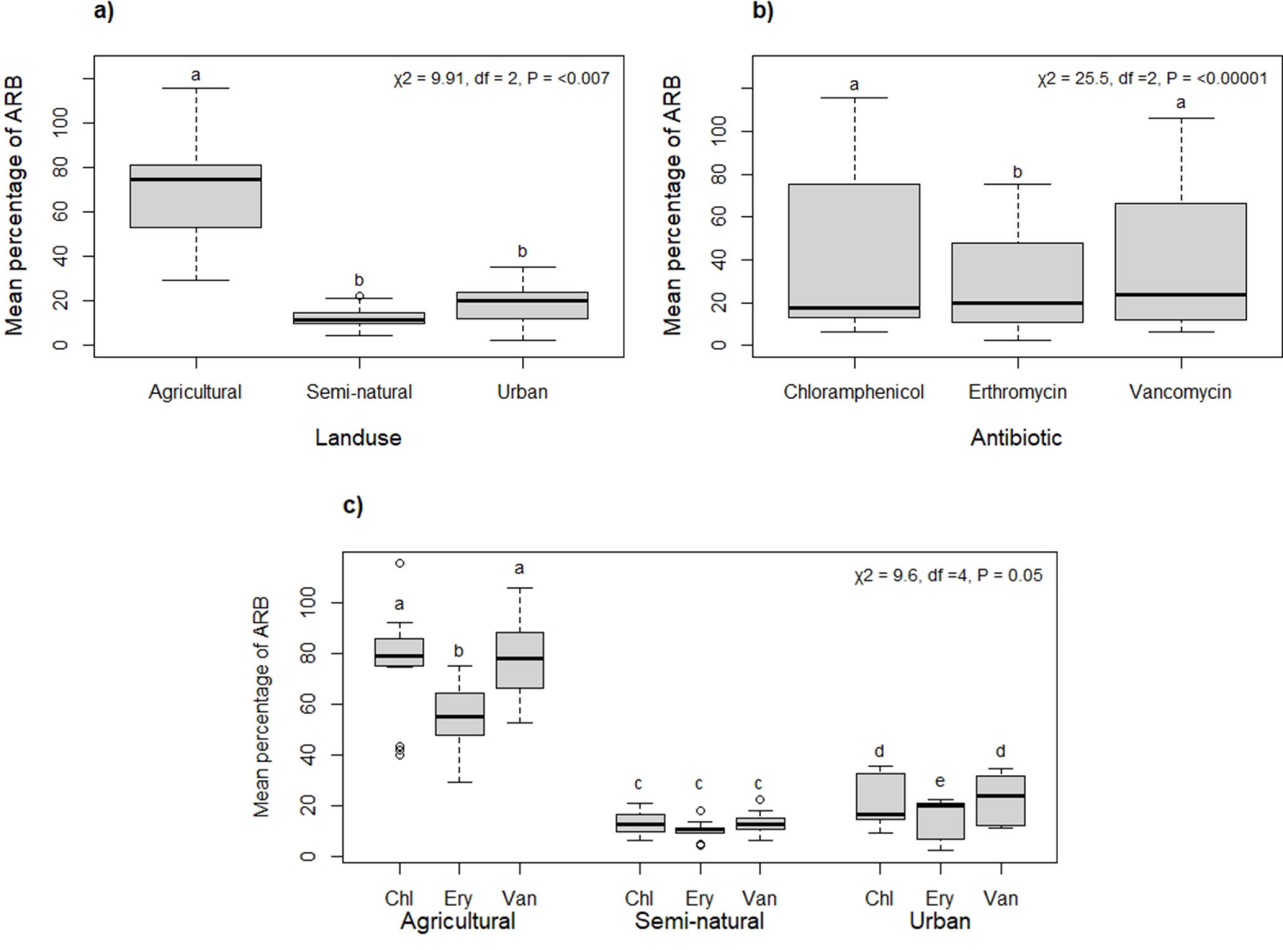
Mean percentage of antibiotic-resistant bacteria (ARB) by (a) land use type, (b) antibiotic type and (c) the interaction between these main terms for the three antibiotics chloramphenicol (Chl), erythromycin (Ery) and vancomycin (Van) at 20 µg ml^−1^. Outcomes of generalized liner mixed models are shown in the top left. Significant differences arising from Tukey post-hoc tests are indicated above each treatment by different letters.

Overall, there was a significant difference in the mean percentage of ARB among the land use types (χ^2^=9.91, df=2, *P* =<0.007); where there was a higher percentage of ARB in agricultural land compared with semi-natural or urban land uses, which showed similar levels of resistance to each other (Fig. 2a). There was a similar mean percentage of ARB for each antibiotic of ~20 % (Fig. 2b), but, when accounting for differences in site location, there was a highly significant difference in the mean percentage of ARB for the antibiotic types (χ^2^=25.5, df=2, *P* =<0.00001) (Fig. 2c). Overall, the mean percentage of ARB was lower for erythromycin than for both vancomycin and chloramphenicol, which did not differ from each other, for both agricultural and urban land use. However, the interaction between land use and antibiotic was marginally significant (χ^2^=9.6, df=4, *P*=0.05). In the semi-natural land use the mean percentage of ARB among all three antibiotics did not differ from each other (Fig. 2c). Trends by individual site can be seen in Fig. S1.

### Agricultural land presents high levels of resistance to high erythromycin concentrations

**Fig. 3.**
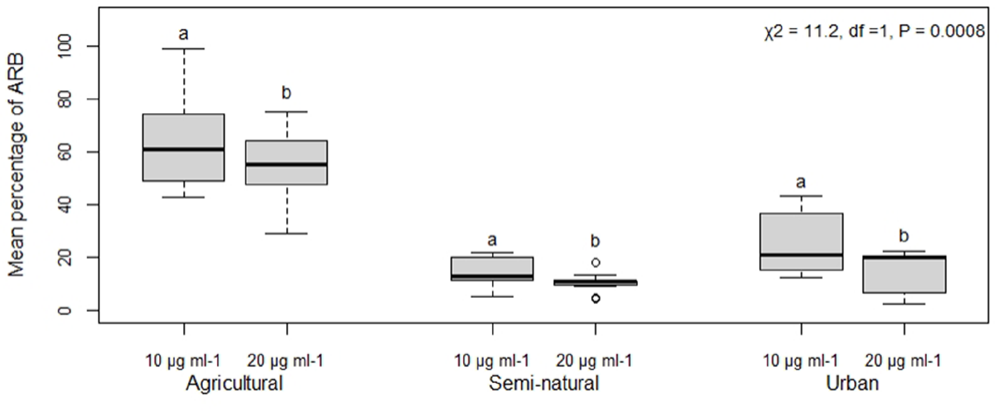
Mean percentage of antibiotic-resistant bacteria (ARB) to different erythromycin concentrations (10 µg ml^−1^ and 20 µg ml^−1^) among the three land use types, agricultural, semi-natural and urban. Outcomes of generalized liner mixed models are shown in the top left. Significant differences between concentrations within each land use, arising from Tukey post-hoc tests, are indicated above each treatment by different letters.

There was a highly significant difference in the mean percentage of ARB between the concentration levels of erythromycin (χ^2^=11.2, df=1, *P*=0.0008), where there were significantly more ARB at 10 µg ml^−1^ than at 20 µg ml^−1^ (Fig. 3). Further, there was a highly significant interaction between erythromycin concentration and land use type (χ^2^=19.4, df=2, *P* =<0.00001), where the difference in ARB between the concentrations was much smaller for agricultural land use soil, compared to semi-natural and urban land use soil (see Fig. S2).

## Discussion

In order to gain an insight into the levels of ARB to three antibiotics in different land use soils in Great Britain, we investigated soils with distinct human activities, i.e. agricultural and urban, and compared these to semi-natural soils. The results from this study show that levels of ARB in agricultural land are significantly higher than those found in either urban or semi-natural land use soils across Great Britain. Similar results have been obtained by previous studies comparing levels of ARB and ARGs between forest and agricultural land in different locations worldwide [51–54]. Our study also showed that there is no significant difference in the levels of ARB present in semi-natural and urban areas across the sites sampled. The level of ARB found in semi-natural and urban areas is likely to reflect the level of antibiotic-producing bacteria present in those soil environments. Naturally occurring antibiotic producers will also carry the genes that confer resistance mechanisms to said molecules. The constant presence of low levels of antibiotics and ARGs in these environments is enough to maintain antibiotic resistance levels within those particular soil populations [55–58]. The levels of ARB found in non-agricultural background soils can provide a good baseline of antibiotic resistance levels in a particular location [59, 60] and therefore can help to determine the impact of management practices on antibiotic resistance within an agricultural system more accurately.

The significantly higher levels of ARB found across agricultural land in this study are likely to be linked to the use of animal slurry in all farms visited. Several studies to date have shown that the use of animal manure in agricultural soil increases the number of ARGs and therefore ARB [61–64], at least for a transient period after their application [65, 66]. The accumulation of ARB in manured agricultural soil depends on the contribution of ARB originated from the gut of the animals, their horizontal gene transfer to soil bacteria, and the selective pressure of antibiotic residues and other chemical stressors present in the soil [9, 64, 67, 68]. Further, although ARGs responsible for macrolides (erythromycin), amphenicols (chloramphenicol) and glycopeptides (vancomycin) have been extensively reported in other soil studies [52, 61, 69–71], to our knowledge, this is the first study that compares the levels of resistance to these three antibiotics within different land use soils in Great Britain.

Interestingly, our results show that resistance levels to vancomycin and chloramphenicol in both agricultural and urban locations are significantly higher than the levels of erythromycin resistance, whilst in semi-natural locations all three antibiotics show similar resistance levels. Vancomycin is considered to be a last-resort antibiotic, and therefore the presence of high vancomycin resistance levels in both agricultural and urban soils in Great Britain not only demonstrates the highly extensive scope of the soil resistome in those locations, but should also be a cause of concern. Similarly high levels of antibiotic resistance to erythromycin, chloramphenicol and vancomycin have previously been reported for both agricultural and urban soils in different countries [69, 72, 73], whilst other studies report negligible levels of resistance to vancomycin compared to those for erythromycin [74]. It is therefore essential to conduct localized antibiotic resistance analyses with respect to specific land uses in order to determine the levels of resistance for different antibiotics in that particular location. In order to determine why the resistance levels to vancomycin and chloramphenicol are significantly higher in the soils with higher levels of human activity sampled in Great Britain in this particular study, further work should aim to identify whether the antibiotic resistance mechanisms of bacteria isolated from different land use soils are due to the acquisition of specific ARGs or multidrug efflux pumps and to establish correlations between resistance mechanisms, soil composition (particularly regarding the levels of metals present) and human activity.

This study also looked at the effect that using different concentrations of erythromycin (10 µg ml^−1^ and 20 µg ml^−1^) had on the number of ARB across the different land use soils. These concentrations were selected based on the lowest minimum inhibitory concentration (MIC) value for erythromycin observed for most bacterial species recorded in the European Committee on Antimicrobial Susceptibility Testing (EUCAST) database [75], ~16 µg ml^−1^. Therefore 10 µg ml^−1^ represents a subinhibitory concentration of erythromycin, whilst 20 µg ml^−1^ represents a concentration above the MIC. The results show that, across the different land use soils, and as we would expect, there are significantly higher levels of bacteria able to grow in the presence of 10 µg ml^−1^ compared to 20 µg ml^−1^ of erythromycin. As with other antibiotics, resistance to erythromycin is not confined to a single mechanism. Several mechanisms of erythromycin resistance have been observed, including reduced penetrability of the cell membrane or active efflux of the molecule to decrease its intracellular concentration, ribosome modification or protection, and drug modification via macrolide phosphotransferases and macrolide esterases [76]. The efficient efflux-mediated mechanisms abundant in soil bacteria can be a source of non-specific multidrug resistance [77, 78], which could be responsible for the increased levels of ARB at subinhibitory concentrations of erythromycin.

It is not surprising that we found that the levels of resistance to different erythromycin concentrations were much less marked in agricultural land soil with respect to the other land use soils. Many findings to date have demonstrated that the use of animal manure in soil increases the reservoir of clinically relevant ARGs [61, 62], and in particular, levels of erythromycin-specific ARGs such as *ermB* are present in high abundance in agricultural soils [52, 69]. While linking an ARG to a specific phenotypic resistance is difficult, especially in the mixed microbial communities common in soil environments, metagenomic analyses to identify the ARGs found in ARB communities able to grow at concentrations of 10 and 20 µg ml^−1^ of erythromycin, respectively, would allow us to detect whether there are differences in the resistance mechanisms present in each ARB population.

In summary, the present study highlights that agricultural land can act as a main reservoir for resistance to clinically relevant antibiotics in Great Britain, compared to urban or semi-natural locations. Understanding the development and spread of antibiotic resistance levels in different land use soils is important in protecting human, animal and ecological health. In this study, we also found that levels of resistance to different antibiotics are dependent on land use. The geographical location and management practices of different countries are likely to have an effect on these differences. Therefore, it is important to conduct localized studies on levels of resistance to specific antibiotics in order to perform appropriate antibiotic resistance risk assessments. Metagenomic analyses to identify the mechanisms driving levels of resistance to different antibiotics in these soil microbial communities would also increase our limited knowledge on how antimicrobial resistance is spread amongst different land use soils.

## Supplementary Data

Supplementary material 1

Supplementary material 2
