# Supplementary material for: Antibiotic resistance levels in soils from urban and rural land uses in Great Britain
Source: Access Microbiol. 2020 Nov 23;3(1):acmi000181. doi: 10.1099/acmi.0.000181 (PMC8115975; doi:10.1099/acmi.0.000181)
Supplement: Supplementary material 1 [file acmi-3-181-s001.pdf]

Supplementary Figures

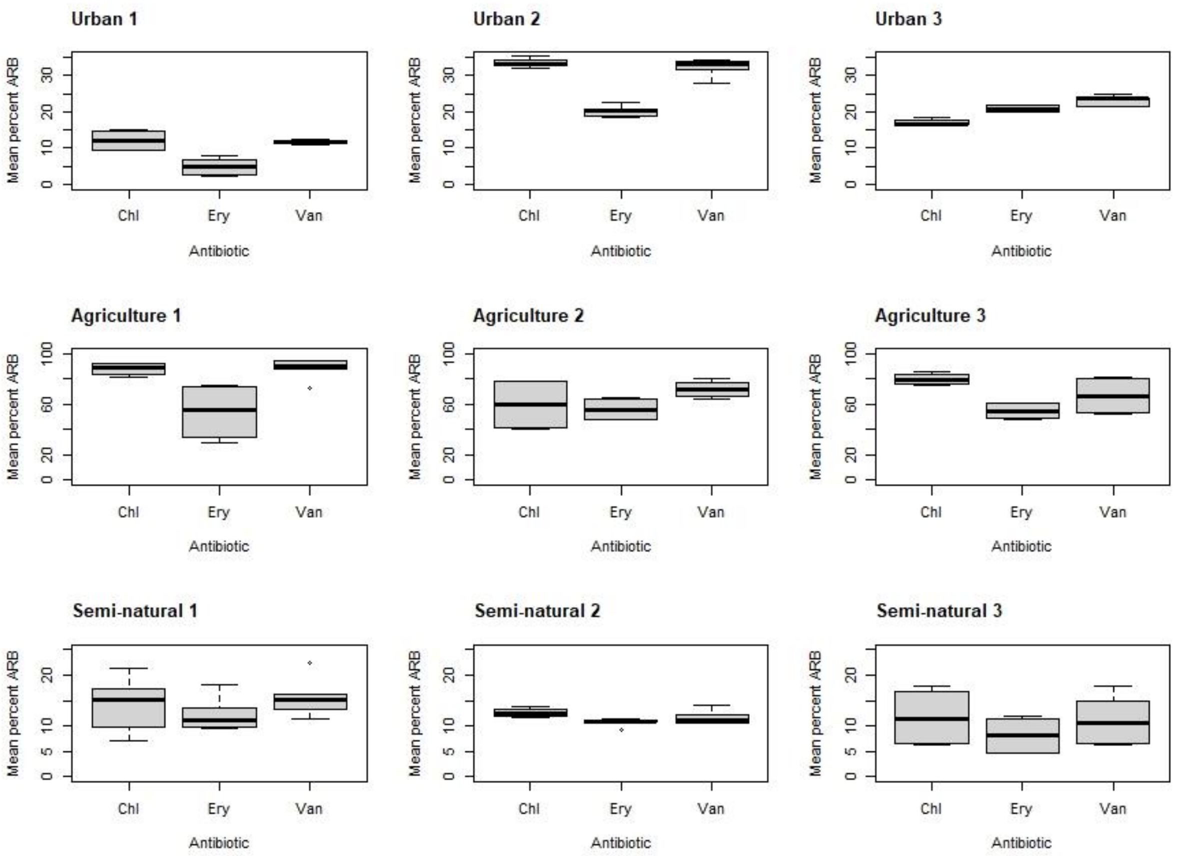

S1 Supplementary Figure 1. Mean percentage of antibiotic resistance bacteria (ARB) for each antibiotic in each site. The antibiotics are Chloramphenicol (Chl), Erythromycin (Ery), Vancomycin (Van), all at 20 µg/ml concentration. To show detail, y-axes have a standardised length within each land-use.

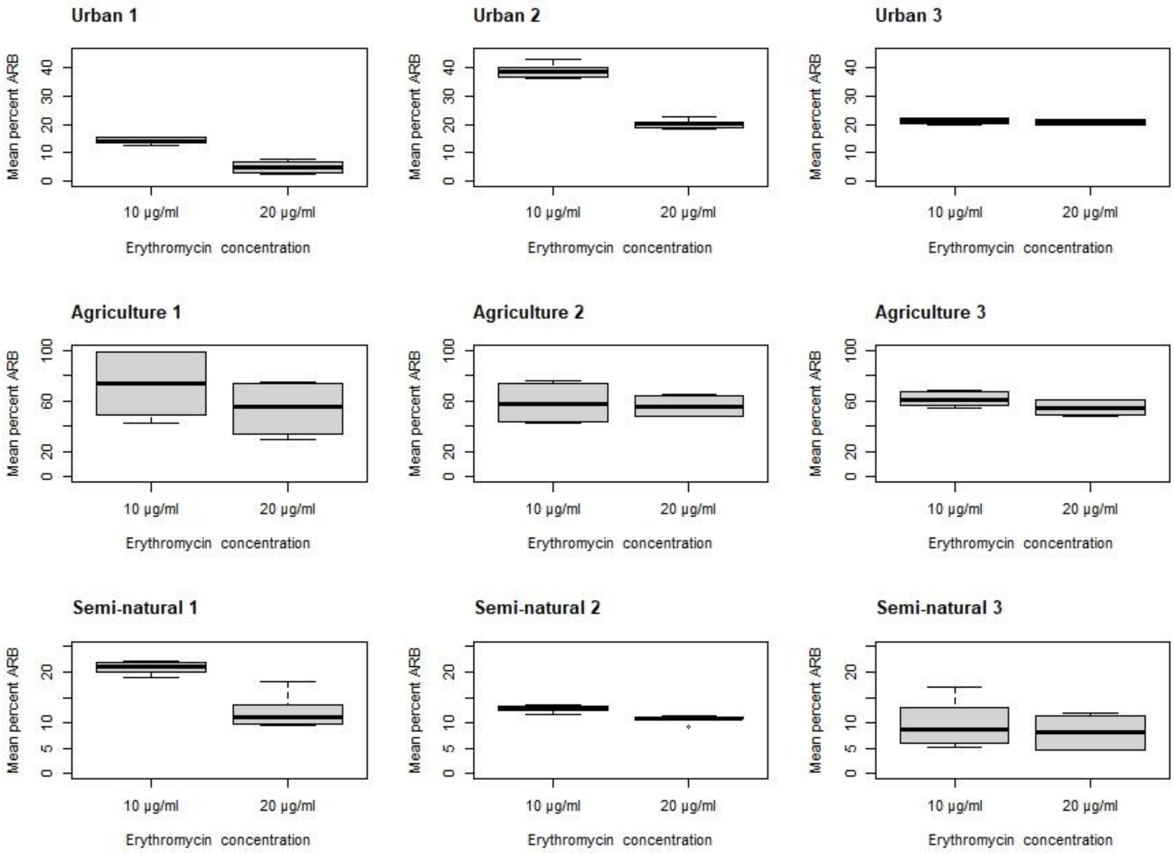

S2 Supplementary Figure 2. Mean percentage of antibiotic resistance bacteria (ARB) for each concentration of erythromycin in each site. To show detail y-axes have a standardised length within each land-use.

S3 Supplementary Table. Raw CFU/ml data obtained for each individual replicate in this study. Site refers to the location where the samples came from. Dilution Factor indicates whether samples were undiluted (0), diluted to 10^{-1} (10), diluted to 10^{-2} (100) or diluted to 10^{-3} (1000). Antibiotic tested indicates the antibiotic tested in that plate. Erythromycin 10 and erythromycin 20 indicate 10 and 20 µg/ml final concentration in the plate respectively. The other antibiotics were all tested at 20 µg/ml final concentration in the plate. Ab present CFU/ml refers to the number of CFU/ml seen in the presence of an antibiotic (indicated in the Antibiotic Tested column) in that plate. Control CFU/ml refers to the number of CFU/ml seen in the absence of antibiotics in that plate to act as a reference of number of CFU/ml from that sample.

The supplementary Table S3 containing the raw data is provided as an excel file.
